# Supplementary material for: Artificial Intelligence vs. Natural Stupidity: Evaluating AI Readiness for the Vietnamese Medical Information System
Source: J Clin Med. 2019 Feb 1;8(2):168. doi: 10.3390/jcm8020168 (PMC6406313; doi:10.3390/jcm8020168)
Supplement: Supplementary file 1 [file jcm-08-00168-s001.zip › R Codes.docx]

Supplementary file for the manuscript titled “Artificial Intelligence vs. Natural Stupidity: Evaluating AI Readiness for the Vietnamese Medical Information System” for submission at the *Journal of Clinical Medicine*.

This file presents the codes for running text mining on the annual reports published by the Vietnamese Ministry of Health from 2012 to 2016.

# Text mining codes

| library(tm)  library(pdftools)  file_location = [INSERT LOCATION]  txt = pdf_text(file_location)  txt_corpus = Corpus(VectorSource(txt))  txt_corpus = tm_map(txt_corpus, tolower)  txt_corpus = tm_map(txt_corpus, removePunctuation)  txt_corpus = tm_map(txt_corpus, stripWhitespace)  txt_corpus = tm_map(txt_corpus, removeWords, "health")  txt_corpus = tm_map(txt_corpus, removeWords, "medical")  txt_corpus = tm_map(txt_corpus, removeWords, "service")  txt_corpus = tm_map(txt_corpus, removeWords, "hospital")  txt_corpus = tm_map(txt_corpus, removeWords, "2012")  txt_corpus = tm_map(txt_corpus, removeWords, "2013")  txt_corpus = tm_map(txt_corpus, removeWords, "2014")  txt_corpus = tm_map(txt_corpus, removeWords, "2015")  txt_corpus = tm_map(txt_corpus, removeWords, "2016")  head(stopwords("en"))  txt_corpus = tm_map(txt_corpus, removeWords, stopwords("en"))  txt_corpus$content  dtm = DocumentTermMatrix(txt_corpus)  dtm = as.matrix(dtm)  dtm = t(dtm)  View(dtm) |
| --- |

# Find the frequency of all words in the report

| number_occurances = rowSums(dtm)  number_occurances = sort(number_occurances, decreasing = TRUE) |
| --- |

# Find the probability of words that appear with “database”, only taking correlation above 30%

| dtm <- TermDocumentMatrix(txt_corpus)  m <- as.matrix(dtm)  v <- sort(rowSums(m),decreasing=TRUE)  d <- data.frame(word = names(v),freq=v)  findAssocs(dtm, terms = "database", corlimit = 0.3) |
| --- |

## Results in 2012

$database
 1252 1342 253 calculating
 0.50 0.50 0.50 0.50
 deepening downward notable prepayment
 0.50 0.50 0.50 0.50
 willingness 092011 toolkit premiums
 0.50 0.50 0.50 0.44
 subsidies premium carry package
 0.36 0.36 0.36 0.35
 recession lacking standardization subsidy
 0.34 0.34 0.34 0.34
 514 discourage basically timelimited
 0.34 0.34 0.34 0.34
 recommends linkages continued establish
 0.34 0.34 0.31 0.31
 move
 0.31

## Results in 2013

$database
 comparable connection expended exploited
 0.84 0.84 0.84 0.84
 justified leaflets magazine summarizing
 0.84 0.84 0.84 0.84
 yearbooks collected records periodic
 0.84 0.78 0.74 0.70
 ethnicity inaccurate done standardizing
 0.69 0.69 0.68 0.64
 incomplete excessive applications evident
 0.63 0.59 0.59 0.59
 fines inefficient statistical gender
 0.59 0.59 0.58 0.56
 surveys data disaggregated summary
 0.53 0.53 0.49 0.48
 posted market accurate enforcement
 0.48 0.48 0.48 0.48
 reports needs intended facilitate
 0.47 0.44 0.42 0.42
 infectious scale therefore information
 0.42 0.41 0.40 0.39
 statistics conducted collecting workload
 0.39 0.39 0.38 0.37
 operation topics economy registration
 0.35 0.33 0.33 0.33
 compiled administrative
 0.32 0.32

## Results in 2014

$database
 planners filling warehouse 288 diversify
 0.63 0.55 0.55 0.55 0.55
 nonhealth coding synthesizing progressed disseminate
 0.55 0.54 0.51 0.51 0.45
 online reports statistical series 792013ndcp
 0.43 0.41 0.40 0.38 0.38
 titles decentralized manually amended consolidation
 0.38 0.38 0.38 0.38 0.38
 forecasts adr formulate 1088qdbyt 1807
 0.38 0.36 0.36 0.36 0.36
 2407 3236 4000 6000 6016
 0.36 0.36 0.36 0.36 0.36
 critiqued finalization library medlib monographs
 0.36 0.36 0.36 0.36 0.36
 searchable versions 032013ttbtc 412011ttbyt 872011ndcp
 0.36 0.36 0.36 0.36 0.36
 criminal dossier solely unify protocols
 0.36 0.36 0.36 0.36 0.35
 information issue dissemination woman completion
 0.33 0.33 0.31 0.31 0.31
 donors analyzing synthesize architecture approve
 0.31 0.31 0.31 0.31 0.31
 directed
 0.31

## Results in 2015

| $database | |  |  |  |  |  |  |
| --- | --- | --- | --- | --- | --- | --- | --- |
| software addresses codes computers | | | | | | | |
| 0.58 0.55 0.55 0.55 | | | | | | | |
| diagnosing digital exchanges hardware | | | | | | | |
| 0.55 0.55 0.55 0.55 | | | | | | | |
| highspeed interfacility itis lan | | | | | | | |
| 0.55 0.55 0.55 0.55 | | | | | | | |
| links servers statistically webbased | | | | | | | |
| 0.55 0.55 0.55 0.55 | | | | | | | |
| mandate preferably unique ministryâ€™s | | | | | | | |
| 0.55 0.55 0.55 0.49 | | | | | | | |
| email identification death applications | | | | | | | |
| 0.49 0.47 0.46 0.44 | | | | | | | |
| personal systems records application | | | | | | | |
| 0.44 0.43 0.43 0.40 | | | | | | | |
| applying exchange breadth gradual | | | | | | | |
| 0.40 0.38 0.38 0.38 | | | | | | | |
| roll dynamics antimicrobials errors | | | | | | | |
| 0.38 0.38 0.38 0.38 | | | | | | | |
| inefficient lowprice medication overprescribing | | | | | | | |
| 0.38 0.38 0.38 0.38 | | | | | | | |
| residues operations coding 523 | | | | | | | |
| 0.38 0.38 0.38 0.38 | | | | | | | |
| modernize sending avoiding impeding | | | | | | | |
| 0.38 0.38 0.38 0.38 | | | | | | | |
| 411 cause use modes | | | | | | | |
| 0.38 0.37 0.37 0.36 | | | | | | | |
| exploited irrational mris registries | | | | | | | |
| 0.36 0.36 0.36 0.36 | | | | | | | |
| information reporting sharing oversight | | | | | | | |
| 0.35 0.35 0.34 0.33 | | | | | | | |
| performance 186 icd10 323 | | | | | | | |
| 0.32 0.31 0.31 0.31 | | | | | | | |
| persists record animal formulating | | | | | | | |
| 0.31 0.31 0.31 0.31 | | | | | | | |
| researchers presence find antibiotic | | | | | | | |
| 0.31 0.31 0.31 0.31 | | | | | | | |
| chain hard wide 412 | | | | | | | |
| 0.31 0.31 0.31 0.31 | | | | | | | |
| prerequisite 144 duplication functioning | | | | | | | |
| 0.31 0.31 0.31 0.31 | | | | | | | |
| hrh patient | | | |  |  |  |  |
| 0.31 0.30 |  |  |  |  |  |  |  |

## Results in 2016

| $database |  |  |  |  |  |
| --- | --- | --- | --- | --- | --- |
| 1998 deliberation expenditure | | | | | |
| 0.55 0.55 0.55 | | | | | |
| flows instrument pilots | | | | | |
| 0.55 0.55 0.55 | | | | | |
| prices7 producing requestâ€\u009d | | | | | |
| 0.55 0.55 0.55 | | | | | |
| subanalysis transmitting â€œvietnam | | | | | |
| 0.55 0.55 0.55 | | | | | |
| 267 273 306 | | | | | |
| 0.55 0.55 0.55 | | | | | |
| 319 349 368 | | | | | |
| 0.55 0.55 0.55 | | | | | |
| 5yhspfiveyear dopdepartment garpglobal | | | | | |
| 0.55 0.55 0.55 | | | | | |
| gatsglobal gdpmgeneral ghedglobal | | | | | |
| 0.55 0.55 0.55 | | | | | |
| ghoglobal gsogeneral mountain | | | | | |
| 0.55 0.55 0.55 | | | | | |
| wasting accounts 240 | | | | | |
| 0.55 0.54 0.49 | | | | | |
| observatory 218 databases | | | | | |
| 0.47 0.45 0.41 | | | | | |
| 195 analyzing version | | | | | |
| 0.38 0.38 0.38 | | | | | |
| refined notes prerequisite | | | | | |
| 0.38 0.38 0.38 | | | | | |
| 36anqcp claims interoperability | | | | | |
| 0.38 0.38 0.38 | | | | | |
| uniform consumables administratively | | | | | |
| 0.38 0.38 0.38 | | | | | |
| subsidies 342 277 | | | | | |
| 0.38 0.38 0.38 | | | | | |
| compiling consolidation maximum | | | | | |
| 0.38 0.38 0.38 | | | | | |
| ntps premium stunting | | | | | |
| 0.38 0.38 0.38 | | | | | |
| underweight 268 281 | | | | | |
| 0.38 0.38 0.38 | | | | | |
| 350 307 malnutrition | | | | | |
| 0.38 0.38 0.38 | | | | | |
| 142 adult 198 | | | | | |
| 0.38 0.38 0.38 | | | | | |
| 303 208 238 | | | | | |
| 0.38 0.38 0.38 | | | | | |
| 259 codes 113 | | | | | |
| 0.38 0.37 0.37 | | | | | |
| institute 165 software | | | | | |
| 0.36 0.32 0.32 | | | | | |
| icd10 fourth disseminated | | | | | |
| 0.31 0.31 0.31 | | | | | |
| reimbursements amenity accurately | | | | | |
| 0.31 0.31 0.31 | | | | | |
| amenities comprehensively output | | | | | |
| 0.31 0.31 0.31 | | | | | |
| useful 275 coastal | | | | | |
| 0.31 0.31 0.31 | | | | | |
| 246 297 130 | | | | | |
| 0.31 0.31 0.31 | | | | | |
| 216 260 329 | | | | | |
| 0.31 0.31 0.31 | | | | | |
| height 145 324 | | | | | |
| 0.31 0.31 0.31 | | | | | |
| 203 207 lists | | | | | |
| 0.31 0.31 0.30 | | | | | |
